# Social cognition in Korsakoff's syndrome: A meta‐analysis

**DOI:** 10.1111/add.70256

**Published:** 2025-11-18

**Authors:** Kyra Wijnen, Willem S. Eikelboom, Yvonne C. M. Rensen, Gwenny T. L. Janssen, Roy P. C. Kessels

**Affiliations:** ^1^ Vincent van Gogh Institute for Psychiatry Centre of Excellence for Korsakoff and Alcohol‐related Cognitive Disorders Venray the Netherlands; ^2^ Donders Institute for Brain, Cognition and Behavior Radboud University Nijmegen the Netherlands; ^3^ Tactus Addiction Care Deventer the Netherlands; ^4^ Radboudumc Alzheimer Center Radboud University Nijmegen Medical Center Nijmegen the Netherlands

**Keywords:** alcohol use disorder, alcohol‐related cognitive disorders, Korsakoff's syndrome, meta‐analysis, neurocognitive disorders, social cognition

## Abstract

**Background and aims:**

Korsakoff's syndrome is an alcohol‐related neurocognitive disorder characterized by episodic memory impairments, apathy, confabulations and poor illness‐insight. This meta‐analysis aimed to estimate mean effect sizes of performance in social cognition in people with Korsakoff's syndrome (KS) compared with controls.

**Method:**

A systematic literature search was conducted in May 2024 to identify research articles that examined social cognition in patients with KS and control groups. Weighted effect sizes (Hedges' *g*) were calculated for the three levels of social cognition: emotion perception, social interpretation and socio‐cognitive integration. There was no restriction on setting. Instruments examining emotion perception (facial emotion recognition and prosody), interpretation (mentalizing, self‐awareness and empathy) and socio‐cognitive integration (moral reasoning and social knowledge) were used in the included studies.

**Results:**

Thirteen studies (*n* = 622; 292 KS, 330 controls) showed that individuals with KS performed statistically significantly worse across all domains of social cognition compared with controls. Large effect sizes were found in emotion perception [*g =* −1.14, 95% confidence interval (CI) = −1.46 to −0.81), *P* < 0.001, I^2^ = 58.5%, 8 studies, *n* = 372], with comparable effect sizes for facial emotion recognition and prosody. In social interpretation (*g =* −0.77, 95% CI = −1.34 to −0.21, *P =* 0.007, I^2^ = 96.6%, 4 studies, *n* = 188), a large effect was found for mentalizing (*g =* −1.05, 95% CI = −1.61 to −0.50, *P* < 0.001, I^2^ = 74.1%; 3 studies, *n* = 120). In socio‐cognitive integration (*g =* −0.74, 95% CI = −1.11 to −0.37, *P* < 0.001, I^2^ = 0%, 3 studies, *n* = 184), social knowledge showed a large effect size (*g =* −0.81, 95% CI = −1.24 to −0.38, *P* < 0.001, 1 study, *n* = 104). Results for empathy (*g =* −0.43, 95% CI = −1.05 to 0.20, *P =* 0.18, 1 study, *n* = 40), self‐awareness (*g =* −0.21, 95% CI = −0.47 to 0.05, *P =* 0.12, 1 study, *n* = 68) and moral reasoning (*k =* 2, *g =* −0.54, 95% CI = −1.28 to 0.19, *P =* 0.15, I^2^ = 0%; 2 studies, *n* = 80) were uncertain, with possible important differences in both directions.

**Conclusions:**

This meta‐analysis shows that people with Korsakoff's syndrome perform statistically significantly worse than controls on socio‐cognitive measures, with the largest effect sizes in the perception and interpretation of social information.

## INTRODUCTION

Social cognition is an umbrella term that refers to the ability to perceive social information, to understand and interpret one's own and others’ thoughts, feelings and intentions, and to respond adequately to this social information [1]. Several authors have argued that social cognition consists of several distinct processes that are inter‐related following a hierarchical structure [1, 2, 3, 4]. Various models distinguish three levels, ranging from lower level automatic and implicit perception of social cues to higher order cognitive abilities: (i) perceiving, (ii) interpreting and (iii) responding to social information [1, 5]. Recently, such a model of social cognition has been updated [6] based on recent consensus‐based definitions for social cognition [7]. For this meta‐analysis the aforementioned Hierarchical Interdependent Taxonomy of Social cognition (HITS) model, developed by Eikelboom *et al*. [6], is adhered to.

The HITS model distinguishes several levels of social cognitive processes. The first level of social cognition describes processes related to the perception of emotions in oneself and in others [6]. Recognizing facial expressions and prosody, interpreting body postures and understanding emotional expressions are included in this level [5]. The second level describes ways in which emotional information is interpreted. An important concept within this level is theory of mind (ToM), defined as ‘the use of folk psychological knowledge and heuristics to think about one's own and other people's mental states’ ([7], p. 2). It allows individuals to predict, anticipate and interpret the behaviors of others. ToM is hypothesized to play a crucial role in facilitating appropriate social interactions. In the HITS model [6], ToM is considered an element of mentalizing: the ability to attribute mental states (e.g. knowledge, intentions, emotions, perception) to oneself and to others [7]. Another concept within the interpretation level is the capacity to understand and experience the emotions of others without conflating them with one's own, referred to as empathy [7, 8]. It enables individuals to resonate with others’ feelings, while maintaining a clear distinction between self and the other. The third and final level of social cognition refers to higher order socio‐cognitive processes that require a combination of cognitive and affective processing [6]. Moral reasoning, for example, allows one to consider and to make decisions about right or wrong, and to think about principles such as justice, harm, fairness and care [9]. The ability to understand and identify social boundaries, referred to as social knowledge, is pivotal in decision‐making in social contexts [10].

In the Diagnostic and Statistical Manual of Mental Disorders, Fifth Edition, Text Revision (DSM‐5‐TR) [11], social cognition is included as one of the core cognitive domains. However, social cognition is often not included in a neuropsychological assessment [3]. Possible explanations include the lack of validated and standardized tests that can be used across different cultures [10], the complexity of the construct [12] and the relatively limited amount of research on social cognition in neurocognitive disorders compared with other cognitive domains.

However, an increasing body of evidence underscores the necessity of assessing social cognition in mental disorders, as social cognitive impairments have been associated with behavioral disturbances such as disinhibition, apathy and reckless behavior [13, 14], as well as poor social participation [15]. Research to date has shown that social cognitive impairments occur in a variety of neurological, neuropsychiatric and developmental disorders [3], such as frontotemporal dementia and Alzheimer's disease dementia [16, 17], psychotic spectrum disorders and bipolar disorder [18], autism spectrum disorder [19, 20], major depression [21], personality disorders [22] and substance use disorders [23].

Alcohol use disorder (AUD) is one of the most studied and most prevalent substance use disorders associated with social cognitive impairments [24]. The ability to recognize emotional facial expressions and interpret body postures are found to be significantly impaired in people with excessive alcohol use, and medium (statistically significant) effects have been found in tasks that aim to measure ToM in people with excessive alcohol use compared with controls [24, 25, 26]. However, studies on social cognition in AUD are limited to individuals with AUD without neurocognitive disorders (NCDs). In those with AUD and NCD, far less research has been done on social cognition. Specifically, the research on social cognition on alcohol‐related major NCD, that is, Korsakoff's syndrome (KS), is an important field to explore.

Korsakoff's syndrome (KS) results from untreated Wernicke encephalopathy (WE), which in turn is caused by thiamine (i.e. vitamin B1) deficiency. It is estimated that 12%–59% of people with AUD develop WE, and an estimated 56%–84% of AUD patients with WE progress to develop KS [27], which highlights the substantial risk of developing this neuropsychiatric disorder in people with AUD. KS is characterized by disproportionate impairments in episodic memory. Confabulations, lack of insight into illness and apathy are also often present in this patient group [27]. Although research to date is limited, several studies have provided evidence for social cognitive impairments in KS. For example, people with KS exhibit a reduced ability to recognize emotions and a significantly lower performance on ToM tasks compared with controls [28, 29, 30]. On the level of social behavior, evidence so far suggests impairment compared with controls [31, 32, 33]. There are at least two plausible explanations for differences that might be observed between patients with KS and controls. First, KS itself may lead to changes in brain function that impair social cognition. Second, the observed differences may reflect pre‐existing characteristics of individuals with AUD who later develop KS, meaning that the group differences result from confounding rather than from a direct effect of the syndrome. However, the research to date has used relatively small samples, has shown mixed results and has used a variety of instruments and theoretical paradigms. Yet, to our knowledge, no review has examined social cognition in people with KS.

Therefore, we conducted a meta‐analysis to summarize performance across different levels of social cognition in patients with KS compared with controls. We expect lower performance in patients with KS. By categorically classifying the existing data using the most up‐to‐date model on social cognition [6], we adopt a unified theory‐driven approach in describing social cognition in KS. The findings of this meta‐analysis will inform clinicians and provide future research directions based on the gaps identified.

## METHODS

This meta‐analysis was pre‐registered with the Open Social Science Framework (OSF) (https://doi.org/10.17605/OSF.IO/5SN2Y), conforms to the PROSPERO format and was conducted in adherence to the PRISMA (Preferred Reporting Items for Systematic reviews and Meta‐Analyses) guidelines. Initially our aim was to perform a scoping review because of the very limited body of evidence we expected to retrieve. However, after completing the systematic search, enough studies were identified to perform a formal meta‐analysis.

### Search strategy

The online electronic databases EMBASE, MEDLINE/Pubmed and PsychINFO were searched from inception to 23 May 2024 (Table S1). Following the publication of a study on social cognition in KS in November 2024, the decision was made to also include this most recent article. In addition, reference lists of the selected studies were manually inspected for potential studies of interest. Finally, members of the author team of this meta‐analysis were consulted to ensure that no relevant studies were missing.

### Study selection

Articles were screened and selected based on the following criteria: (i) clinical diagnosis of KS, preferably based on an internationally recognized classification system, such as the DSM, the International Classification of Diseases (ICD) or conventional consensus criteria [34]; (ii) the inclusion of a control group without cognitive impairments (i.e. no diagnoses of neurocognitive disorders) and with no history or current diagnosis of a substance use disorder, including AUD; (iii) the assessment of social cognitive levels [i.e. tasks measuring emotion perception (both facial emotion recognition and prosody), interpretation (mentalizing, empathy, attribution style and self‐awareness) and socio‐cognitive integration (moral reasoning and social knowledge)]; (iv) the provision of necessary information to perform a meta‐analysis (e.g. means, standard deviations, median range, detailed graphs).

No restriction was placed on publication date or setting. In the case of longitudinal data, only baseline data were used. Two independent reviewers (K.W. and W.S.E) screened titles and abstracts and subsequently inspected the full articles for eligibility. Discrepancies were discussed and a consensus was reached.

### Data extraction

Data from each paper were extracted in duplicate (K.W. and W.S.E.). In articles where statistical information was missing, the principal investigators were contacted.

### Quality assessment

Two independent reviewers (K.W. and W.S.E.) evaluated the quality of each study using an adjusted quality assessment tool for observational studies from the National Heart, Lung and Blood Institute [35]. This tool includes 14 quality criteria that cover the methodology and study population characteristics. However, as we only used observational cross‐sectional studies in this review and exposure was defined as a dichotomous variable (diagnosis of KS, yes/no), four of its items (6, 7, 8 and 13) were not relevant for our study and were not evaluated (Table S2).

### Data synthesis and analysis

For this meta‐analysis we compared people diagnosed with KS with cognitively unimpaired controls. For studies that reported more than one outcome measure of a specific task, a single effect size (Hedges’ *g*) was calculated by averaging multiple effect sizes within the task.

For the studies that reported medians and interquartile ranges, means and standard deviations were manually estimated using established formulas [36]. For the study that reported means and standard errors, standard deviations were derived [37]. If studies only reported effect sizes, but no means and standard deviations, the reported effect sizes were converted to Hedges’ *g* using conventional formulas. For all analyses, a negative effect size indicates poorer performance (i.e. more social cognitive problems) in the KS group compared with the control group.

The used instruments of the included studies were categorized under (sub)levels by comparing the measurement aims of the instruments with the HITS model [6]. For several studies the construct measured by the instruments used in the included studies differed from the construct defined in the HITS model [29, 38]. This reflects the large heterogeneity in definitions and terminologies used in the field of social cognition [6]. To limit this heterogeneity, we re‐evaluated all instruments in terms of the HITS model. For example, the cognitive empathy and affective empathy scales of the Interpersonal Reactivity Index (IRI) were considered as ‘mentalizing’ and ‘empathy’ following the definitions of the HITS model and Quesque *et al*. [7]. Similarly, ToM measures such as the Sally–Anne and faux pas tests were also categorized under mentalizing. Two studies used experimental tasks that were designed for the specific study [38, 39]. In those studies, the intended measurement aims were inferred from the task description and subsequently categorized under (sub)levels of the HITS model.

The following subgroup analysis was selected beforehand: performance on social cognition tasks was analyzed according to level (emotion perception, social interpretation and socio‐cognitive integration). For each study, the included instruments were allocated to sublevels in accordance with the model of Eikelboom *et al*. [6]. For emotion perception the sublevels were prosody (e.g. Montreal Affective Voices Battery, MAVB [40]) and facial emotion recognition (e.g. Emotion Recognition Task, ERT [29, 41]). For interpretation, mentalizing (e.g. Sally–Anne test [42]), empathy (e.g. empathic concern subscore of the IRI [43]) and self‐awareness were included. Finally, for socio‐cognitive integration, the included sublevels were moral reasoning (e.g. Moral Behavior Inventory, MBI [44]) and social knowledge (e.g. Social Norms Questionnaire, SNQ [45]). Other concepts of the model, such as attribution style and emotion regulation, were not researched in the selected studies and were therefore not included in the analyses.

For most analyses of the (sub)levels, the total scores of the instruments were used. In some cases, the methodology underlying the calculation of specific scores is worth mentioning. First, in two studies [28, 46] an average total score of the instruments was used as these instruments were categorized under the same level (e.g. Sally–Anne and faux pas, which are both categorized under social interpretation). In the sublevel analyses, the total scores were used for these separate instruments for one of these studies [46]. Second, two studies reported on the same participant sample; therefore, an average total score of the instruments used was included in the level analysis for emotion perception. For the sublevel analyses, the prosody subscore of the Emotion Detection Task was combined with the total score of the MAVB [47, 48]. Finally, two instruments assessed multiple sublevels: the Emotion Detection Task includes both a score for facial emotion recognition and prosody [47], and the IRI yielded both a mentalizing and empathy score [49]. For the two latter studies, both a total score (for level analyses) and subscores (for the sublevel analyses) were therefore used.

Heterogeneity was assessed with the *I* ^2^ statistic and tested using the Cochran's *Q*‐test. The *I* ^2^ statistic is an appraisal of the consistency of the effect sizes. Rough guidelines are as follows: 0%–40% suggests no heterogeneity, 30%–60% suggests moderate heterogeneity, 50%–90% suggests substantial heterogeneity and 75%–100% suggests considerable heterogeneity across studies [50].

Funnel plot asymmetry was used as an indicator for publication bias. Egger's regression tests were used to test for funnel plot asymmetry for (sub)levels with more than five studies included in the analyses [51, 52]. If any of these tests were indicative for publication bias, the trim‐and‐fill method was used to estimate the number of missing studies and to recompute the summary statistics based on complete data.

All analyses were conducted using IBM® SPSS® 29.0.2.0 (IBM, Armonk, NY, USA), which was also used to make the figures. A random‐effects model was deemed appropriate considering differences in experimental methodology across studies and variation in outcome measures.

## RESULTS

### Characteristics of included studies

**FIGURE 1:**
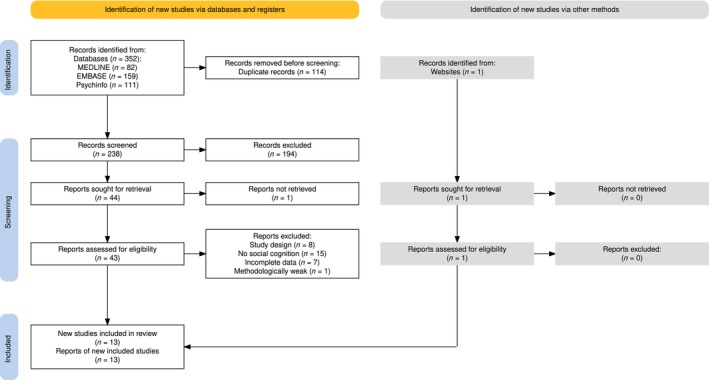
PRISMA (Preferred Reporting Items for Systematic reviews and Meta‐Analyses) flow chart of the literature search [53].

**FIGURE 2:**
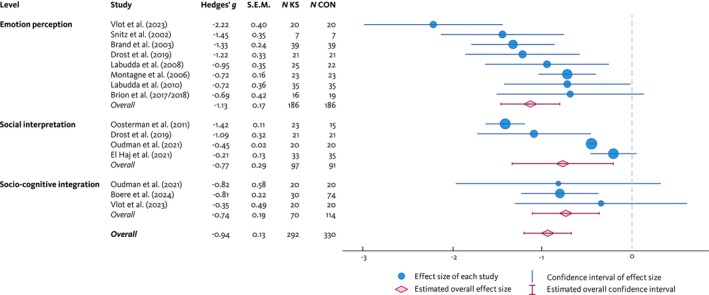
Forest plot showing the effects sizes of the individual studies and the overall effect sizes of the levels of social cognition (random‐effects model, test of between‐subgroup homogeneity, *Q* = 2.80, d.f. = 2, *P* = 0.25).

A total of 239 unique articles were found and screened for eligibility (Figure 1). The full texts of 45 studies were retrieved, 13 of which were included, with a total of *n =* 622 participants. Of all participants, 292 were diagnosed with KS (46.9%) and 330 (53.1%) participants were controls. All controls had no diagnosis of AUD and had no NCD (Figure 2).

Three authors sent additional statistical details upon request, while no additional information was received for one article. In one other study, graphs were measured to retrieve the mean and SD, as tables were missing and attempts to contact the author remained unsuccessful.

Most studies made use of performance measures (e.g. the ERT, MBI, Sally–Anne test), while one study used the IRI, a self‐report measure, as the main outcome. Nine studies used standardized instruments, four used experimental paradigms that were either specifically designed for the study or derived from validated instruments and adapted for the study (Table 1).

**TABLE 1:** Sample characteristics of the included studies.

Study	*n*	Diagnostic criteria KS	Mean (SD) age, years	SC tests used	SC level	Setting	GR M : F	Mean education level (years or Verhage)	Abstinence duration	Country
Labudda *et al*., 2008^a^	25 KS, 22 C	ICD‐10 & DSM‐IV	55.84 (6.53) KS, 53.45 (9.22) C	AWT, EPT	Emotion perception	Long‐term care	15 : 10 KS, 13 : 9 C	Years: 9.76 (2.17) KS 9.50 (1.90) C	Not reported	DE
Labudda *et al*., 2010^a^	35 KS, 35 C	ICD‐10 & DSM‐IV	57.37 (7.34) KS, 54.20 (6.46) C	EPT, SCT	Emotion perception	Long‐term care	22 : 13 KS 21 : 14 C	Years: 9.34 (1.77) KS, 8.74 (1.2) C	Not reported	DE
Brion *et al*., 2017	16 KS, 17 AUD, 19 C	DSM‐V	51.44 (8.56) KS, 50.71 (8.23) AUD, 51.84 (7.19) C	Emotion detection task (unimodal, congruent and non‐congruent)	Emotion perception	Long‐term care	10 : 6 KS 8 : 9 AUD 9 : 10 C	Years: 11.13 (2.21) KS, 13.59 (2.71) AUD, 16.95 (4.21) C	265 days	BE
Brion *et al*., 2018	16 KS, 17 AUD, 19 C	DSM‐V	51.4 (8.5) KS, 50.7 (8.2) AUD, 51.8 (7.2) C	MAVB	Emotion perception	Long‐term care	10 : 6 KS, 8 : 9 AUD, 9 : 10 C	Years: 11.1 (KS), 13.6 (AUD) 16.9 (HC)	>76–812 days	BE
Montagne *et al*., 2006	23 KS, 23 C	DSM‐IV	53.0 KS, 54.12 C	ERT, BFRT (Short Form)	Emotion perception	Mental healthcare	16 : 7 both groups	Verhage: 4.3 KS, 4.55 C	Not reported	NL
Snitz *et al*., 2002^b^	7 KS, 7 C	Not reported	57.86 KS, 58.57 C	Tübingen Affect Battery	Emotion perception	Long‐term care	All male	Not reported	Not reported	DE
Brand *et al*., 2003^c^	39 KS, 39 C	ICD‐10 & DSM‐IV	56.77 (6.49) KS, 59.74 (7.55) C	AWT	Emotion perception	Long‐term care	23 : 16 KS 22 : 17 HC	Years: 27 (<9), 6 (10), 6 (>11) KS 22 (<9), 13 (10), 4 (>11) C	Not reported	DE
Drost *et al*., 2019	21 KS, 21 C	DSM‐V	59.9 KS, 68.7 C	Sally–Anne test, faux‐pas test, ERT	Emotion perception & social interpretation	Long‐term care	17 : 4 both groups	Verhage: 4.2 KS, 5.0 HC	>1 year	NL
Oosterman *et al*., 2011	23 KS, 15 C	DSM‐IV	52.1 KS, 52.4 C	Story comprehension task: to assess perspective taking abilities	Social interpretation	Mental healthcare	17 : 6 KS, 11 : 4 HC	Verhage: 4.2 KS 4.9 C	>2 months	NL
El Haj *et al*., 2021	33 KS, 35 C	DSM‐IV	57.48 (5.43) KS, 56.17 (5.36) C	Rating two own autobiographical memories on subjective experience and autobiographical specificity	Social interpretation	Not reported	15 : 17 KS, 16 : 19 C	Years: 8.76 (3.32) KS 9.80 (4.32) C	Not reported	FR
Oudman *et al*., 2021	20 KS, 20 C	DSM‐V	61.75 (7.83) KS, 64.10 (9.44) C	Moral dilemmas (So Moral), two subscales IRI	Social interpretation & socio‐cognitive integration	Long‐term care	12 : 8 both groups	Verhage: 4.50 KS 4.95 C	Not reported	NL
Boere *et al*., 2024	30 KS, 10 ARD, 74 C	DSM‐V	65.9 KS, 67.3 ARD, 64.7 C	SNQ	Socio‐cognitive integration	Long‐term care	20 : 10 KS, 6 : 4 ARD, 44 : 30 C	Years: 10.7 KS, 9.8 ARD, 11.7 C	>1 year	NL
Vlot *et al*., 2023	20 KS, 20 C	DSM‐V	61.70 KS, 61.55 C	MBI, ERT, Delinquency QS	Emotion perception & socio‐cognitive integration	Long‐term care	14 : 6 both groups	Verhage: 4.45 KS, 4.65 C	>6 months	NL

Abbreviations: AUD = alcohol use disorder; AWT = Affective Word Test; BFRT = Benton Facial Recognition Test; C = control; DSM = Diagnostic and Statistical Manual of Mental Disorders; EPT = Emotional Picture Task; ERT = Emotion Recognition Task; F = female; FAB = Frontal Assessment Battery; GR = gender ratio; ICD = International Classification of Diseases and Health Related Problems; IRI = Interpersonal Reactivity Index; KS = Korsakoff syndrome; M = male; MAVB = Montreal Affective Voices Battery; MBI = Moral Behavior Inventory; SCT = Semantic Classification Task; SNQ = Social Norms Questionnaire.

^a^ Median and interquartile range.

^b^ Standard error, measured manually.

^c^ Education level categorized in absolute number of participants per year.

### Study quality

All of the included studies had an overall rating of fair quality (*k* = 13) (Table S2). Duration of alcohol use, comorbidity and duration of abstinence were not consistently reported in the individual studies.

### Results for the three levels of social cognition

#### Emotion perception

We found lower performance in people with KS compared with controls on measures of emotion perception, with a large effect size (*g =* −1.14, 95% CI = –1.46 to −0.81, *P < 0*.001, *I* ^2^ = 58.5%, eight studies, *n* = 407). Within the level of emotion perception a large effect size was found for both facial emotion recognition (*g =* −1.13, 95% CI = –1.62 to −0.64, *P* < 0.001, *I* ^2^ = 67%, six studies, *n* = 280) and prosody (*g =* −1.09, 95% CI = –1.54 to −0.65, *P* < 0.001, *I* ^2^ = 57.7%, five studies, *n* = 174).

#### Social interpretation

We found lower performance in people with KS compared with controls on measures of social interpretation, with a large effect size (*g =* −0.77, 95% CI = –1.34 to −0.21, *P =* 0.007, *I* ^2^ = 96.6%, four studies, *N* = 188). Within this level, a large effect size was found for mentalizing (*g =* −1.05, 95% CI = –1.61 to −0.50, *P* < 0.001, *I* ^2^ = 74.1%, three studies, *n* = 120). For empathy the findings were uncertain, with confidence interval values suggesting the possibility of significant differences in both directions (*g =* −0.43, 95% CI = –1.05 to 0.20, *P =* 0.18, one study, *n* = 40). A small effect size was found for self‐awareness (*g =* −0.21, 95% CI = –0.47 to 0.05, *P =* 0.12, one study, *n* = 68). For empathy and self‐awareness, the *I* ^*2*^ statistic could not be computed as there was only one study included for these respective sublevels.

#### Socio‐cognitive integration

Lower performance was found in people with KS compared with controls on scales that assess socio‐cognitive integration, with a large effect size (*g =* −0.74, 95% CI = –1.11 to −0.37, *P* < 0.001, *I* ^2^ = 0%, three studies, *n* = 184). Within this level, a large effect was found for social knowledge (*g =* −0.81, 95% CI = –1.24 to −0.38, *P* < 0.001, one study, *n* = 104). For moral reasoning, the effect size was medium, with confidence interval values suggesting the possibility of significant difference in both directions (*g =* −0.54, 95% CI = –1.28 to 0.19, *P =* 0.15, *I* ^2^ = 0%, two studies, *n* = 80). For social knowledge, the *I* ^*2*^ statistic could not be computed as there was only one study included in this sublevel; for forest plots and funnel plots of the (sub)levels, see Figures S1 and S2). See Figure 2 for a forest plot on the (overall) effect sizes of the individual studies and levels of social cognition.

### Publication bias

The Egger's regression test and visual inspection of funnel plots revealed no evidence of publication bias in the meta‐analyses for (sub)levels that included more than five studies (Table 2).

**TABLE 2:** Analyses and sub‐analyses of the (sub)levels of social cognition in patients with Korsakoff's syndrome compared with controls.

Level	Studies	n	Hedges’ *g* (95% CI)	*Z‐*statistic	*P*‐value	*Q*‐statistic	*P*‐value *Q*‐statistic	*I* ^2^ statistic	Egger's regression‐based test (95% CI)
**Emotion perception**	8	372	−1.14 (−1.46, −0.81)	−6.759	<0.001	17.324	0.015	58.5	−0.63 (−2.17, 0.92)
Facial emotion recognition	6	280	−1.13 (−1.62, −0.64)	−4.518	<0.001	14.245	0.014	67.0	−0.58 (−2.35, 1.19)
Prosody^b^	5	174	−1.09 (−1.54, −0.65)	−4.849	<0.001	6.924	<0.074	57.7	N/A
**Social interpretation** ^b^	4	188	−0.77 (−1.34, −0.21)	−2.680	0.007	78.881	<0.001	96.6	N/A
Mentalizing^b^	2	120	−1.05 (−1.61, −0.50)	−3.736	<0.001	8.153	0.017	74.1	N/A
Empathy^a^ ^,^ ^b^	1	40	−0.43 (−1.05, 0.20)	−1.348	0.18	N/A	N/A	N/A	N/A
Self‐awareness^a^ ^,^ ^b^	1	68	−0.21 (−0.47, 0.05)	−1.578	0.12	N/A	N/A	N/A	N/A
**Socio‐cognitive integration** ^b^	3	184	−0.74 (−1.11, −0.37)	−3.896	<0.001	0.757	0.685	0	N/A
Moral reasoning^b^	2	80	−0.54 (−1.28, 0.19)	−1.443	0.15	0.391	0.532	0	N/A
Social knowledge^a^ ^,^ ^b^	1	104	−0.81 (−1.24, −0.38)	−3.669	<0.001	N/A	N/A	N/A	N/A
**Overall**	13	657	−0.94 (−1.21, −0.68)	−7.003	<0.001	128.576	<0.001	87.0	−0.70 (−1,29, −0.12)

^a^ Heterogeneity for these sublevels could not be computed as there was only one study included.

^b^ Analysis on publication bias (Egger's regression based test) could not be computed owing to the low number of studies.

## DISCUSSION

This is the first meta‐analysis comparing performance across various levels of social cognition in people with alcoholic KS compared with controls with neither cognitive impairments nor AUD. Our findings evidently show that performance in emotion recognition, mentalizing and social knowledge is poorer in people with alcoholic KS compared with controls, with medium to large effect sizes.

A previous meta‐analysis by Bora and Zorlu [24] on social cognition in AUD also showed lower performance in patients with AUD, with medium effect sizes in emotion perception and ToM. The results of the current meta‐analysis for patients with KS suggest a similar pattern of impairments, albeit with larger effect sizes (but with moderate to substantial heterogeneity) in both emotion perception and social interpretation. At the level of social interpretation we specifically found a large effect size for ToM, which is part of mentalizing in the updated model we used [6]. These findings suggest that the profile of deficits in social cognition are not specific to KS, as individuals with AUD without NCD also show reduced performance in social cognition. However, the effect sizes in patients with KS are larger than those in individuals with AUD for the (sub)levels of emotion perception and mentalizing. This could be explained by several factors, which we will discuss below.

First, patients with KS may have had a more severe drinking history than the AUD group that was included in the meta‐analysis by Bora and Zorlu [24]. While there is some evidence of a dose–response relationship between the amount and duration of alcohol consumption and cognitive dysfunction [54, 55], to date, evidence on such a relationship with deficits in specific cognitive domains, including social cognitive dysfunction, is lacking [56]. Also, none of the studies included in the current meta‐analysis provided data on the duration and intensity of the drinking history.

Second, the brain damage in the diencephalon associated with KS [27] may also have resulted in more severe social cognitive dysfunction than in people with AUD only. For instance, thalamic atrophy is known to be more severe in those with KS than in those with AUD without NCD [27, 57]. The thalamus may be an important brain region associated with social cognition; for example, it was found that a reciprocal connection between the ventral midline thalamic area and the medial prefrontal cortex (mPFC) is essential for emotion recognition [58]. Future studies should relate performance in social cognitive measures with structural and functional neuroimaging in patients with KS to unravel the neural underpinning of social cognition deficits in KS [59, 60].

Thirdly, the larger effect sizes for social cognitive impairment in KS compared with AUD might be associated with the profound impairments in other cognitive domains that are typical for KS, but not for AUD, notably in (episodic) memory and executive functioning [61]. Tasks developed to measure social cognition often require several inferential steps to reach an answer, which appeals to (working) memory (i.e. memorizing instructions and stories) and executive functioning. For example, a study showed that performance on a perspective‐taking task in the KS group decreased as the task complexity increased, which suggests that performance was mediated by the level of executive function [38].

Finally, as social cognitive impairment is described in a variety of mental disorders, the prevalence of psychiatric comorbidity may be an important factor. In people with KS, mood disorders as well as psychotic and personality disorders are frequently observed [62]. These conditions are all associated with deficits in social cognition [22, 47]. Yet, while mood disorders are more prevalent in people with KS compared with people with AUD, psychotic disorders and personality disorders are similarly prevalent or may even be less common [63, 64]. All in all, future research should directly compare the performance of individuals with AUD without NCD and individuals with KS on social cognition tasks, taking drinking history, deficits in non‐social cognitive domains and psychiatric comorbidities into account.

Our meta‐analysis showed consistent medium to large effect sizes for performance in emotion recognition, mentalizing and social knowledge. However, the effect sizes were uncertain for empathy, self‐awareness and moral reasoning, while deficits in these sublevels have been reported in people with AUD [65, 66]. As patients with KS also have a history of AUD, it is expected that patients with KS show similar impairments in these domains. Therefore, the difference in the findings might be explained by the methodological challenges that affect the measurement of social cognition. Examples of such challenges include poor psychometric quality, ceiling or floor effects, and inconsistencies in the definitions of constructs. For example, definitions of the construct ‘empathy’ have previously been inconsistent and subject to ambiguity [67], whereas other constructs such as ‘emotion perception’ are more established [6]. The lack of consensus on certain definitions in social cognition has complicated the development of instruments with good construct validity. Consequently, assessing higher order processes of social cognition remains challenging as its measurement often entails a complex interplay of constructs.

Additionally, it should be noted that analyses of the sublevels that rendered no significant findings were based on a very limited number of studies. Although findings are not statistically significant, point estimates were sometimes large and included large effect sizes in the respective confidence intervals. Results for these sublevels are therefore insensitive, with confidence intervals including the possibility of important effects in both directions. More data would be needed to understand how outcomes differ between people with KS and controls.

The findings of this meta‐analysis may have important implications. Our findings clearly show that social cognitive functioning is lowered in people with KS compared to controls without cognitive impairment and without AUD. As the effect sizes found are large and are present in several social cognitive levels, they are likely to be clinically relevant. It is therefore recommended that tests of social cognition should be included in the standard neuropsychological assessment of people suspected of having KS and/or alcohol‐related cognitive impairments. Furthermore, social cognitive impairments should be taken into account in behavioral treatments, given that such impairments have been associated with behavioral, emotional and therapy adherence‐related problems [68, 69, 70]. Non‐pharmacological treatments targeted towards social cognition have been described previously in various neuropsychiatric disorders (for a review, see [71]). Moreover, interventions aimed at ameliorating social cognitive impairments have been successfully implemented as part of the treatment for people with acquired brain injury [72, 73]. However, such an intervention has never been studied in people with KS and people with other alcohol‐related cognitive disorders. For these populations, such an intervention should take into account the (sometimes severe) cognitive impairments (e.g. memory impairments or impairments in executive functions) and the compromised learning capacity of these patient groups [61].

There is a need for the continued development of validated instruments to assess all (sub)levels of social cognition, and to incorporate these into standard neuropsychological assessment. While several validated tests are available for facial emotion recognition and mentalizing, far fewer validated instruments are available to assess empathy, and higher order socio‐cognitive functions, such as moral reasoning, social knowledge and emotion regulation. This may also be reflected in the low number of studies available that have studied these sublevels in KS. Therefore, future studies should focus particularly on novel instruments to study empathy and higher order socio‐cognitive functions in KS. In addition to social cognition, social behavior needs to be explored further. As social behavior is observable and may reflect real‐world social cognition deficits, observational instruments (based on informant reports) and self‐report measures offer valuable additional ecologically valid information to pen‐and‐paper social cognition tasks [74, 75]. A promising instrument is the Observable Social Cognition: A Rating Scale (OSCARS), which has been validated for people with schizophrenia [18, 74], but not for people with KS. Future studies should relate measures of social cognition to measures of social behavior to gain a deeper level of understanding on social functioning in everyday life.

The strengths of this study include the systematic subdivision of the different levels and concepts of social cognition, in accordance with the HITS model [6], which allowed for a theory‐driven analysis. The limitations of this meta‐analysis primarily stem from the limited number of studies included and the heterogeneity observed across these studies. Consequently, a reliable analysis on publication bias was not feasible for some (sub)levels because of the small number of studies. Similarly, a meta regression was not possible owing to the small numbers of studies and the lack of sufficient data on variables such as duration of alcohol abuse, psychiatric comorbidity and duration of abstinence. The heterogeneity across studies varied greatly. This can be explained by the large variety of tests and paradigms used, as well as the use of different theoretical frameworks. While this reflects the current availability of studies on this topic, it complicates analyses on effect sizes and heterogeneity for sublevels such as empathy, self‐awareness and moral reasoning. Finally, as all studies were conducted in Western European countries, focused on a specific age group (albeit aligned with the epidemiology [76]) and other confounding variables such as demographic factors were not consistently reported, the findings cannot be confidently generalized to other ethnicities, cultures and regions [77].

## CONCLUSION

This meta‐analysis shows that people with KS perform more poorly than controls on social cognition measures, with moderate to large effect sizes. The largest effect sizes were found in the perception and interpretation of social information. Compared with previous findings on people with AUD, the profile of deficits is similar, but the impairments in social cognition appear to be more profound in people with KS. While more research is needed to identify causal relationships, our findings may guide future approaches to diagnostics and treatment options for social cognition in KS.

## AUTHOR CONTRIBUTIONS

**Kyra Wijnen:** Conceptualization (lead); data curation (lead); formal analysis (lead); investigation (lead); methodology (equal); project administration (lead); visualization (equal); writing—original draft (lead). **Willem S. Eikelboom:** Conceptualization (supporting); formal analysis (equal); funding acquisition (supporting); investigation (supporting); methodology (equal); supervision (equal); writing—review and editing (equal). **Yvonne C. M. Rensen:** Investigation (supporting); supervision (supporting); writing—review and editing (equal). **Gwenny T. L. Janssen:** Investigation (supporting); supervision (supporting); writing—review and editing (equal). **Roy P. C. Kessels:** Formal analysis (supporting); investigation (supporting); methodology (supporting); project administration (supporting); supervision (equal); visualization (equal); writing—review and editing (equal).

## DECLARATION OF INTERESTS

The authors declare that they do not have any competing interests.

## STUDY REGISTRATION

Registration details can be found at https://doi.org/10.17605/OSF.IO/5SN2Y.

## Supporting information


**Table S1.** Search strategy literature research.
**Table S2.** Study quality assessment.

**Figure S1.** Forest plot estimated effect sizes per sublevel of social cognition.

**Figure S2.** Funnel plots for meta‐analysis on social cognition levels.

## Data Availability

All scripts and output files are available at https://doi.org/10.17605/OSF.IO/5SQ62.
