# Supplementary material for: Social cognition in Korsakoff's syndrome: A meta‐analysis
Source: Addiction. 2025 Nov 18;121(4):765–76. doi: 10.1111/add.70256 (PMC12980293; doi:10.1111/add.70256)
Supplement: Supplementary file 1 — Table S1. Search strategy literature research. Table S2. Study quality assessment. [file ADD-121-765-s001.docx]

**Social cognition in Korsakoff’s syndrome: a meta-analysis**

Running title: Social cognition in Korsakoff’s syndrome

Kyra Wijnen^1^, Willem S. Eikelboom^1,2^, Yvonne C. M. Rensen^1,2^, Gwenny T. L. Janssen^1^, & Roy P. C. Kessels^1,2,3,4^

^1^ Vincent van Gogh Institute for Psychiatry, Centre of Excellence for Korsakoff and Alcohol-related Cognitive Disorders, 5803 DN Venray

^2^ Radboud University, Donders Institute for Brain, Cognition and Behaviour, 6525 GD Nijmegen, The Netherlands

^3^ Tactus Addiction Care, Deventer, The Netherlands

^4^ Radboud University Nijmegen Medical Center, Radboudumc Alzheimer Center, Nijmegen, The Netherlands

Corresponding author: Roy P.C. Kessels, roy.kessels@donders.ru.nl

**Supplemental materials**

**eTable 1. Search strategy literature research**

**eTable 2. Study quality assessment**

**eFigure 1. Forest plot estimated effect sizes per sublevel of social cognition**

**eFigure 2. Funnel plots for meta-analysis on social cognition levels**

**eTable 1. Search strategy literature research**

| MEDLINE from inception until May 23, 2024  1 KS  ("Wernicke-Korsakoff" OR "Wernicke Encephalopathy" OR "Korsako*" OR "Alcohol Amnestic Disorder" OR "Alcohol Dementia").mp.  2 Social cognition  ("Social cogniti*" OR "social behav*" OR "empathy" OR "Theory of Mind" OR "Social Perception" OR "emotion*" OR "mentalizing" OR "mentalising" OR "mindreading" OR "mental state attribution" OR "attribution").mp.  1 AND 2  N=82 |
| --- |
| EMBASE from inception until May 23, 2024  1 KS  ("Wernicke-Korsakoff" OR "Wernicke Encephalopathy" OR "Korsako*" OR "Alcohol Amnestic Disorder" OR "Alcohol Dementia")  2 Social cognition  ("Social cogniti*" OR "social behav*" OR "empathy" OR "Theory of Mind" OR "Social Perception" OR "emotion*" OR "mentalizing" OR "mentalising" OR "mindreading" OR "mental state attribution" OR "attribution")  1 AND 2  N=159 |
| PsychINFO from inception until May 23, 2024  1 KS  ("Wernicke-Korsakoff" OR "Wernicke Encephalopathy" OR "Korsako*" OR "Alcohol Amnestic Disorder" OR "Alcohol Dementia")  2 Social cognition  ("Social cogniti*" OR "social behav*" OR "empathy" OR "Theory of Mind" OR "Social Perception" OR "emotion*" OR "mentalizing" OR "mentalising" OR "mindreading" OR "mental state attribution" OR "attribution")  1 AND 2  N=111 |

**eTable 2. Study quality assessment based on the criteria outlined in [36]**

| Author, year | 1 | 2 | 3 | 4 | 5 | 6 | 7 | 8 | 9 | 10 | 11 | 12 | 13 | 14 | Overall quality |
| --- | --- | --- | --- | --- | --- | --- | --- | --- | --- | --- | --- | --- | --- | --- | --- |
| Brand et al., 2003 | Yes | Yes | NR | Yes | No | NA | NA | NA | Yes | NA | Yes | No | NA | Yes | Fair |
| Brion et al., 2017 | Yes | Yes | NR | Yes | No | Na | NA | NA | Yes | NA | Yes | No | NA | Yes | Fair |
| Brion et al., 2018 | Yes | Yes | NR | Yes | No | NA | NA | NA | Yes | NA | Yes | No | NA | Yes | Fair |
| Drost et al., 2019 | Yes | Yes | NR | Yes | No | NA | NA | NA | Yes | NA | Yes | No | NA | Yes | Fair |
| El Haj et al., 2021 | Yes | Yes | NR | Yes | No | NA | NA | NA | Yes | NA | No | No | NA | Yes | Fair |
| Labudda et al., 2008 | Yes | Yes | NR | Yes | No | NA | NA | NA | Yes | NA | No | No | NA | Yes | Fair |
| Labudda et al., 2010 | Yes | Yes | NR | Yes | No | NA | NA | NA | Yes | NA | No | No | NA | Yes | Fair |
| Montagne, 2006 | Yes | Yes | NR | Yes | No | NA | NA | NA | Yes | NA | Yes | No | NA | No | Fair |
| Oosterman et al., 2011 | Yes | Yes | NR | Yes | No | NA | NA | NA | Yes | NA | No | No | NA | Yes | Fair |
| Oudman et al., 2021 | Yes | Yes | NR | Yes | No | NA | NA | NA | Yes | NA | Yes | No | NA | Yes | Fair |
| Snitz et al., 2002 | Yes | Yes | NR | Yes | No | NA | NA | NA | Yes | NA | Yes | No | NA | Yes | Fair |
| Vlot et al., 2023 | Yes | Yes | NR | Yes | No | NA | NA | NA | Yes | NA | Yes | No | NA | No | Fair |
| Boere et al., 2024 | Yes | Yes | NR | Yes | No | NA | NA | NA | Yes | NA | Yes | NR | NA | Yes | Fair |
