# Supplementary figures and images for: Social cognition in Korsakoff's syndrome: A meta‐analysis
Source: Addiction. 2025 Nov 18;121(4):765–76. doi: 10.1111/add.70256 (PMC12980293; doi:10.1111/add.70256)

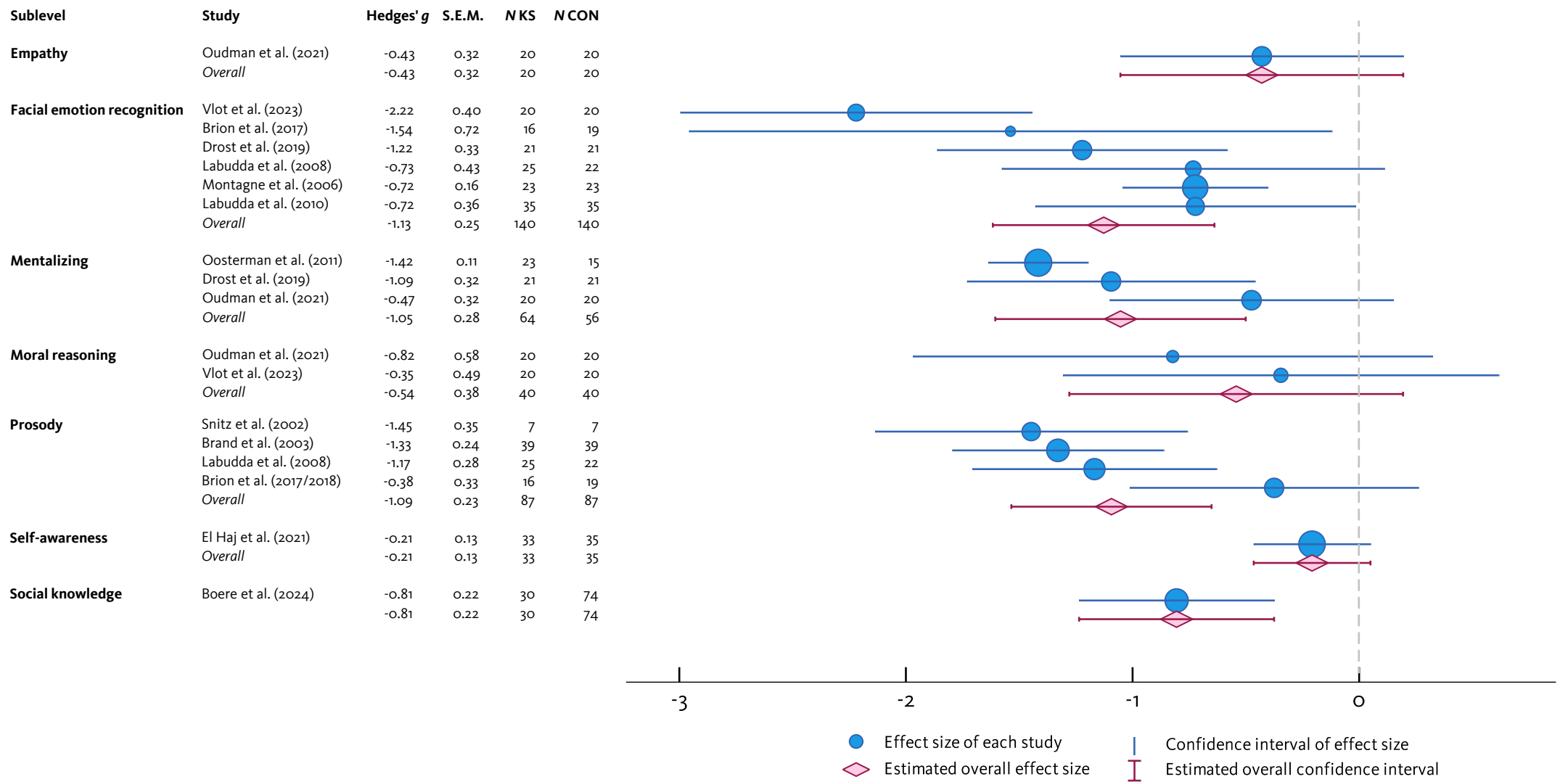

Supplement: Supplementary file 2 — Figure S1. Forest plot estimated effect sizes per sublevel of social cognition. [file ADD-121-765-s003.pdf]
