# Supplementary material for: Social cognition in Korsakoff's syndrome: A meta‐analysis
Source: Addiction. 2025 Nov 18;121(4):765–76. doi: 10.1111/add.70256 (PMC12980293; doi:10.1111/add.70256)
Supplement: Supplementary file 3 — Figure S2. Funnel plots for meta‐analysis on social cognition levels. [file ADD-121-765-s002.pdf]

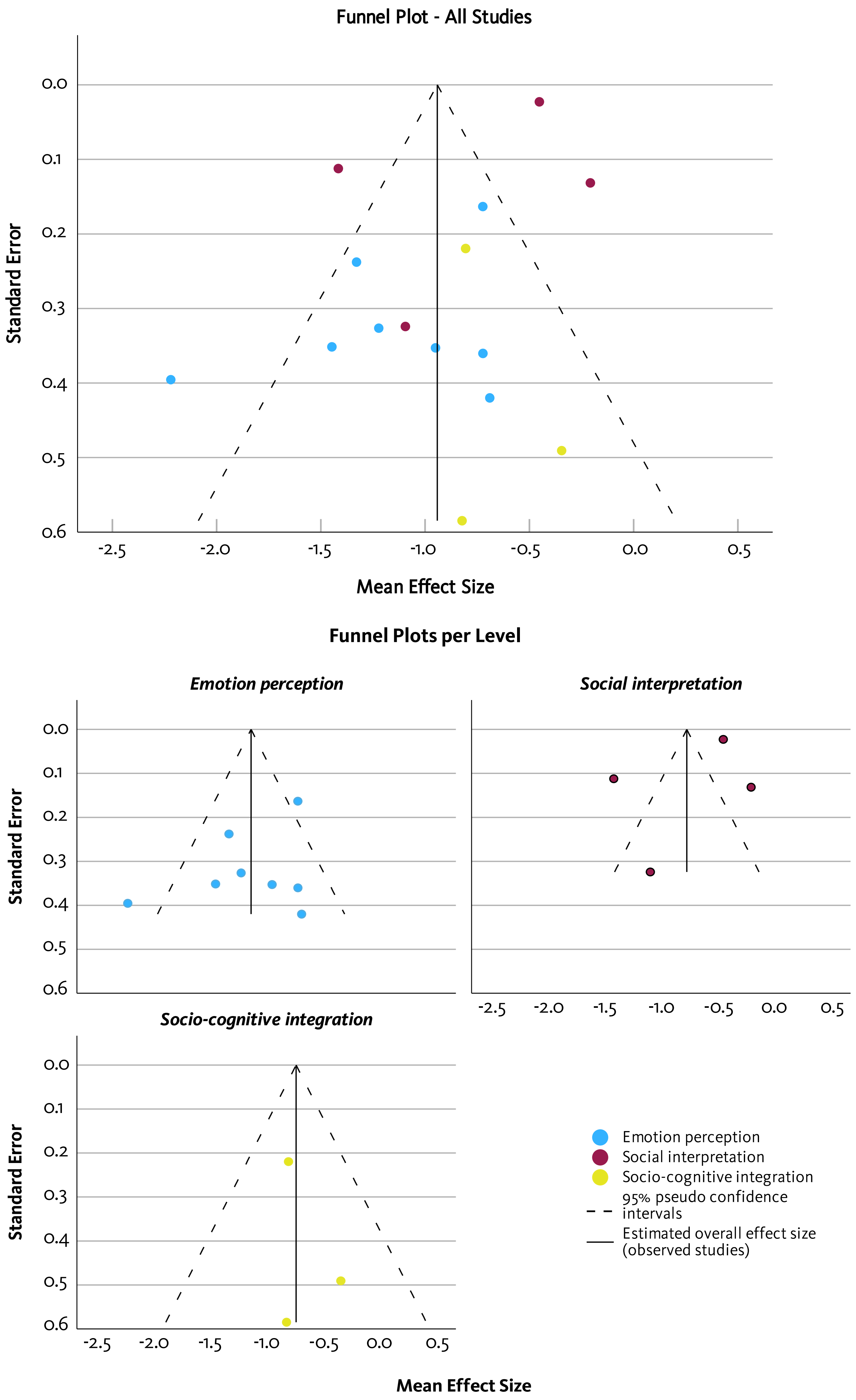

Funnel Plot - All Studies
Standard Error
0.0
0.1
0.2
0.3
0.4
0.5
0.6
-2.5
-2.0
-1.5
-1.0
-0.5
0.0
0.5
Mean Effect Size
Funnel Plots per Level
Emotion perception
Social interpretation
Socio-cognitive integration
Emotion perception
Social interpretation
Socio-cognitive integration
95% pseudo confidence intervals
Estimated overall effect size (observed studies)
